# Fronto-striatal dopamine D2 receptor availability is associated with cognitive variability in older individuals with low dopamine integrity

**DOI:** 10.1038/s41598-021-00106-y

**Published:** 2021-10-26

**Authors:** Saana M. Korkki, Goran Papenberg, Nina Karalija, Douglas D. Garrett, Katrine Riklund, Martin Lövdén, Ulman Lindenberger, Lars Nyberg, Lars Bäckman

**Affiliations:** 1 Aging Research Center, Karolinska Institute and Stockholm University, Stockholm, Sweden; 2 Department of Radiation Sciences, Diagnostic Radiology, Umeå University, Umeå, Sweden; 3 Umeå Center for Functional Brain Imaging, Umeå University, Umeå, Sweden; 4 Center for Lifespan Psychology, Max Planck Institute for Human Development, Berlin, Germany; 5 Max Planck UCL Centre for Computational Psychiatry and Ageing Research, Berlin, Germany; 6 Department of Psychology, University of Gothenburg, Gothenburg, Sweden; 7 Department of Integrative Medical Biology, Umeå University, Umeå, Sweden

**Keywords:** Neuroscience, Psychology

## Abstract

Within-person, moment-to-moment, variability in behavior increases with advancing adult age, potentially reflecting the influence of reduced structural and neurochemical brain integrity, especially that of the dopaminergic system. We examined the role of dopamine D2 receptor (D2DR) availability, grey-, and white-matter integrity, for between-person differences in cognitive variability in a large sample of healthy older adults (n = 181; 64–68 years) from the Cognition, Brain, and Aging (COBRA) study. Intra-individual variability (IIV) in cognition was measured as across-trial variability in participants’ response times for tasks assessing perceptual speed and working memory, as well as for a control task of motor speed. Across the whole sample, no associations of D2DR availability, or grey- and white-matter integrity, to IIV were observed. However, within-person variability in cognition was increased in two subgroups of individuals displaying low mean-level cognitive performance, one of which was characterized by low subcortical and cortical D2DR availability. In this latter group, fronto-striatal D2DR availability correlated negatively with within-person variability in cognition. This finding suggests that the influence of D2DR availability on cognitive variability may be more easily disclosed among individuals with low dopamine-system integrity, highlighting the benefits of large-scale studies for delineating heterogeneity in brain-behavior associations in older age.

## Introduction

In addition to mean-level decreases in many cognitive domains, older age is characterized by increased variability, or inconsistency, of behavioral performance^1,2^. Age-related increases in within-person variability have most commonly been observed for measures of reaction time (RT) in various perceptual-motor and cognitive tasks^3–8^, but also for performance accuracy, such as variability in the accuracy of memory retrieval^9–11^. Importantly, such increases are typically seen after controlling for differences in mean performance, suggesting that they are not a mere product of age-related decreases in average performance levels^3,5,6^. Indeed, between-person differences in mean RT and intra-individual variability (IIV) in RT have been found to predict unique variance in cognitive performance cross-sectionally^3,12^, with longitudinal evidence further suggesting utility of IIV in predicting cognitive decline in healthy aging^13–15^, as well as mild cognitive impairment and dementia^16,17^, beyond measures of mean performance^13,16^.

These observations have led to proposals that IIV may serve as a sensitive index of neural integrity, supported by neuroimaging studies linking multiple indices of brain integrity to within-person variability in behavior^2,18^. For instance, individual differences in grey- and white-matter volume^19–21^, white-matter microstructure^22^ and hyperintensity burden^23,24^ have been related to between-person differences in IIV, with many studies emphasizing the importance of structural integrity of the prefrontal cortex^21,23,25,26^. On the functional level, stability of behavioral performance has been linked to task-based activation of fronto-parietal and striatal regions^27–30^ that support goal-oriented behavior and cognitive control^31–34^. These findings are consistent with cognitive accounts attributing behavioral variability to fluctuations in the efficiency of executive control processes^8,35^.

Further, the neurotransmitter dopamine (DA) may play a critical role in maintaining consistent behavioral performance^2,18,36,37^. In addition to older age, increased IIV has been observed in several clinical conditions affecting the DA system, including Parkinson’s disease^38,39^, attention-deficit hyperactivity disorder^40^, and schizophrenia^41,42^. Moreover, variation in DA-related genes is associated with differences in IIV among healthy individuals^43–45^. An association between DA and behavioral variability is consistent with the involvement of DA neurotransmission in modulating the dynamics^46–50^ and fidelity^51–54^ of neural signalling, balancing between network stability and flexibility^34^. Indeed, prior positron emission tomography (PET) studies have linked DA receptor availability to within-person variability in cognition in middle and older age^55,56^. More specifically, DA D2-like receptor (D2DR) availability in the hippocampus, orbitofrontal cortex, and anterior cingulate was linked to IIV in executive function and episodic memory in a group of middle-aged adults^56^. Further, MacDonald et al.^55^ found age-related reductions in D1 receptor availability in the cingulo-fronto-parietal network to mediate age-related increases in IIV observed during an attentional interference task.

Thus, prior studies suggest that multiple markers of reduced brain integrity are related to increased within-person variability in behavior, especially highlighting a role for reduced DA-system integrity in aging. However, most studies have focused on one imaging modality, not enabling simultaneous assessment of the importance of different candidate factors. Moreover, previous PET studies examining relationships between DA and IIV have relied on small samples, making it difficult to evaluate potential between-person differences in the association between DA and IIV in healthy aging. In particular, consistent with the pattern observed for DA and mean-level cognitive performance^33^, it is possible that such an association may be non-linear^57^, or perhaps detectable only in certain subgroups of older individuals who exhibit reduced DA system integrity below a critical threshold^58,59^.

We assessed associations between multiple measures of brain integrity (D2DR availability, grey-matter volume, white-matter microstructure, and white-matter hyperintensity burden) and behavioral IIV in a large sample of healthy older adults. Participants (n = 181; 64–68 years) were recruited from the Cognition, Brain, and Aging (COBRA) study. Within-person variability in cognition was measured as the intra-individual standard deviation (ISD) of participants’ response times in tasks of perceptual speed and working memory, as well as for a control task of motor speed. Prior findings from the COBRA study suggest within-sample heterogeneity in the association between DA and mean-level cognitive performance^60^. Specifically, the COBRA sample has been found to comprise three distinct subgroups of individuals displaying (a) high cognitive performance and high D2DR availability, (b) low cognitive performance and low D2DR availability, and (c) low cognitive performance, but high D2DR availability. Here, we leveraged these prior results to examine whether the DA-behavior associations observed for mean-level cognitive performance extend to within-person variability in cognition. In particular, we expected greater levels of IIV in the subgroups of individuals displaying low cognitive performance, consistent with the close relationship of these two aspects of performance in aging^12^. Furthermore, we probed the role of individual differences in brain integrity to any such increases observed, with the prediction that reductions in D2DR availability may play a particular role in increased IIV for individuals with low DA-system integrity^59^.

## Methods

Study design, recruitment procedure, imaging protocols, and cognitive and lifestyle batteries for the COBRA study have been reported in detail elsewhere^61^. Here, we only describe methodological details relevant to the current work. The study was approved by the Swedish Ethical Review Authority (Umeå, Sweden; registration number: 2012-57-31M). All methods were performed in accordance with the relevant guidelines and regulations. All participants provided written informed consent prior to testing.

### Participants

The sample consisted of 181 healthy older individuals (64–68 years of age; mean age = 66.2; SD = 1.2; 81 women), randomly selected from the population register of Umeå, a city in northern Sweden. Exclusion criteria included brain pathology and trauma, impaired cognitive functioning (Mini Mental State Examination score < 27), and conditions that could bias the brain measurements or cognitive performance, or preclude imaging. Mean education in the sample was 13.3 years (SD = 3.5), mean body-mass index 26.1 (SD = 3.5), mean systolic blood pressure 142 (SD = 17), and mean diastolic blood pressure 85 (SD = 10). The sample is representative of the healthy target population in Umeå^61^.

### Behavioral measures

From the broad cognitive test battery of the COBRA study^61^, the present analyses focused on tasks that allowed for the assessment of IIV through collection of trials-specific RTs. Specifically, the tasks included here consisted of three indicators of perceptual speed, as well as an n-back working memory task completed during a functional magnetic resonance imaging (fMRI) session. Analyses of the in-scanner n-back task were restricted to the 1-back condition, as examination of participants’ response time distributions indicated this condition to be suitable for the current analyses. For higher loads, the response time limitations imposed by the task (max response time of 1.5 s) resulted in a truncated distribution of RTs across trials, limiting the reliability of IIV estimates in these conditions (see Supplementary material). Moreover, an additional task of motor speed was included to assess whether any observed relationships were specific to within-person variability in cognition, rather than reflecting an influence of motor variability.

The perceptual-speed tasks included numerical, letter, and figural-comparison tasks. In letter comparison, two four-letter strings (comprised of letters A-Z) appeared on a computer screen, and participants’ task was to decide whether or not the two strings were identical. Similarly, in number comparison, participants judged whether two four-number strings (comprised of numbers 1–9) were identical or different. In figure comparison, participants were presented with two novel figures (“fribbles”; courtesy of Michael J. Tarr, Brown University, Providence, RI, USA, http://www.tarrlab.org), and judged whether or not they were identical. In each of these tasks, non-identical stimuli pairs differed only in terms of one item (i.e., one letter, or number), or one figure constituent. Each perceptual-speed task consisted of two blocks of 40 trials (ISI: 0.5 s). Participants responded with their left and right index finger, using two labelled buttons on a keyboard. The task was self-paced, but with each trial timing out after 5 s if no response was given. In the case of no response, the same trial was repeated. Participants were instructed to respond as quickly and accurately as possible and completed a practice block before each of the tasks.

The in-scanner numerical n-back task consisted of three load conditions: 1-back, 2-back, and 3-back. In each condition, a sequence of single numbers was presented on the screen (stimulus duration: 1.5 s, ISI: 0.5 s), and participants were asked to report whether the current number matched the number presented *n*-back (i.e., 1-, 2- or 3-back) in the sequence, by pressing one of two adjacent buttons with their index or middle finger. Nine blocks of 10 items each were completed for each load, in a randomized order. The trial sequence was the same across participants. At the start of each task block, the load condition was indicated by a label on the screen. Practice trials were completed prior the main task. As described above, the current analyses focus on the 1-back condition.

Motor speed was assessed with a finger-tapping task, in which maximum finger-tapping frequency was measured^62,63^. When performing this task, the index finger of one hand was placed on a color-marked key, and the number of taps for each hand during 25 s was registered. Before registering tapping frequencies, one training session per hand was performed.

### Image acquisition

Magnetic resonance imaging (MRI) was performed with a 3 T Discovery MR 750 scanner (General Electric), equipped with a 32-channel phased-array head coil, and PET with a Discovery PET/CT 690 scanner (General Electric) and the ligand ^11^C-raclopride.

#### Structural MRI

T1-weighted high-resolution anatomical images were acquired with a 3D fast spoiled gradient-echo sequence. Each image comprised 176 slices with a thickness of 1 mm (TR = 8.2 ms; TE = 3.2 ms; flip angle = 12°; field of view = 25 × 25 cm). White-matter microstructure was assessed using diffusion tensor imaging (DTI). Diffusion-weighted images were acquired by a spin-echo-planar T2-weighted sequence, with 3 repetitions and 32 independent directions. The total slice number was 64, with TR = 8000 ms, TE = 84.4 ms, flip angle = 90°, field of view = 25 × 25 cm, and *b* = 1000 s/mm^2^. Furthermore, a fluid-attenuated inversion recovery (FLAIR) sequence was acquired to assess white-matter hyperintensities (WMH). The total number of slices for the FLAIR sequence was 48, slice thickness = 3 mm, TE = 120 ms, TR = 8000 ms, and field of view = 24 × 24 cm.

#### PET

PET was performed during rest conditions following an intravenous bolus injection of 250 MBq ^11^C-raclopride. Preceding the injection, a low-dose helical CT scan (20 mA, 120 kV, 0.8 s/revolution) was obtained for attenuation correction. Following the injection, a 55-min 18-frame dynamic PET scan was acquired (9 × 120 s + 3 × 180 s + 3 × 260 s + 3 × 300 s). Attenuation- and decay-corrected images (47 slices, field of view = 25 cm, 256 × 256-pixel transaxial images, voxel size = 0.977 × 0.977 × 3.27 mm^3^) were reconstructed with the iterative point-spread function ordered subset maximization (PSF-OSEM) algorithm VUE Point HD-SharpIR (GE^64^; 6 iterations, 24 subsets, 3.0 mm post filtering), yielding full width at half maximum (FWHM) of 3.2mm^65^. Head movements during the imaging session were minimized with individually fitted thermoplastic masks attached to the bed surface.

### Image preprocessing and analyses

#### Grey-matter volumes

Subcortical brain structures were delineated with the Freesurfer 6.0 software^66^, and cortical parcellation was performed according to the Desikan-Killiany atlas^67^. The regions of interest (ROI) for the volumetric, as well as for the PET data, included the striatum and the prefrontal cortex, motivated by prior evidence highlighting the importance of fronto-striatal circuits for cognitive control and executive function^31,33^, processes thought to underpin within-person variability in behavioral performance^8,35^. Striatal volume was computed as the mean of putamen and caudate volumes, and frontal volume as the mean of the superior, middle and inferior frontal gyrus, respectively (left and right volumes were averaged for each region, and the regional volumes z-transformed). The average striatal and frontal volumes were adjusted for total intracranial volume (TIV) via the method of covariance^68^ (adjusted volume = raw volume – *b*(TIV – mean TIV), where *b* is the slope of the regression of the regional volume on TIV).

#### White-matter microstructure

DTI analysis was performed using the FMRIB Software Library (FSL) package (http://www.fmrib.ox.ac.uk/fsl) and Tract-Based Spatial Statistics (TBSS), as part of the FMRIB software package. The three subject-specific diffusion acquisitions were concatenated in time followed by eddy-current correction. Accordingly, the b-matrix was reoriented based on the transformation matrix^69^. The first volume within the averaged volume that did not have a gradient applied (i.e., the first *b* = 0) was used to generate a binary brain mask with the Brain Extraction Tool^70^. Finally, DTIfit was used to fit a diffusion tensor to each voxel included in the brain mask/space, yielding voxel-wise maps of fractional anisotropy (FA). Using the TBSS processing stream, all subject-specific FA maps were nonlinearly normalized to standard space and then fed into a skeletonize program to make a skeleton of common white-matter tracts across all subjects. Mean diffusivity (MD) images were processed based on the results of the processing of the FA images, yielding individual MD skeletons. We computed the average FA and MD across two association tracts that connect the frontal cortex to the parietal, temporal and occipital cortices (superior longitudinal fasciculus; SLF, superior fronto-occipital fasciculus; SFOF), as well as for the genu of the corpus callosum (CC) connecting the two frontal hemispheres with reference to JHU ICBM-DTI-81 white-matter labels^71^. Composite FA and MD measures were then created as the average across these three tracts (tract-specific measures first z-transformed).

#### White-matter hyperintensities

WMHs were segmented by the lesion-growth algorithm^72^, as implemented in the LST toolbox, version 2.0.14 (www.statisticalmodelling.de/lst.html) for SPM12. First, the algorithm segmented the T1 images into the three main tissue classes (cerebrospinal fluid, grey matter, and white matter). This information was combined with the coregistered FLAIR intensities to calculate lesion-belief maps. By thresholding these maps with a pre-chosen initial threshold (κ = 0.3, defined by visual inspection), an initial binary lesion map was obtained. This map was then grown along hyperintense neighboring voxels in the FLAIR image, resulting in a lesion-probability map that, after thresholding (50%), yielded a binary map of lesions from which the total volume (cm^3^) and number of lesions per individual were obtained.

#### D2DR availability

D2DR availability was determined by calculating ^11^C-raclopride binding potential (BP_ND_) within the FreeSurfer-segmented regions of interest. In brief, the PET images were motion-corrected and co-registered with the corresponding T1-weighted structural image using the Statistical Parametric Mapping software (SPM12, https://www.fil.ion.ucl.ac.uk/spm/). Time-activity curves for the reported regions were used to calculate BP_ND_ using the Logan method^73^. The cerebellar grey matter was used as a reference region, due to negligible D2DR expression^74^. For each region, left and right hemisphere D2DR BP_ND_ were averaged. Composite striatal and frontal measures were then computed as the average of the putamen and caudate, and the average of the superior, middle and inferior frontal gyrus, respectively (regional BP_ND_ measures z-transformed; see Supplementary Table S1 for mean BP_ND_ in each subregion). Although suitability of the low-affinity ligand ^11^C-raclopride for the assessment of cortical D2DR availability remains debated^75–77^, increasing evidence suggests reliability and validity of ^11^C-raclopride BP_ND_ for measurement of D2DR availability beyond the striatum. Specifically, extrastriatal ^11^C-raclopride BP_ND_ has been observed to have good test–retest reliability^78,79^, to vary according to anatomical and functional organization of the DA system^80^, to correspond to post-mortem and high-affinity ligand estimates of D2DR availability^79,80^, as well as to associate with between-person differences in cognitive performance^81–83^. Further, extrastriatal ^11^C-raclopride displacement following pharmacological^84–86^ and cognitive^87,88^ manipulation of DA release has been reported, supporting the view that extrastriatal ^11^C-raclopride BP_ND_ constitutes a meaningful marker of DA neurotransmission.

### Subgroup classification

The identification of subgroups was conducted previously and is described in detail in Lövdén et al^60^. To recapitulate, latent-profile analysis (LPA), a probabilistic Gaussian mixture-modeling approach for identifying hidden subgroups, was performed to identify latent classes explaining multivariate associations between cognitive performance (indexed by composite scores of episodic memory, working memory, and perceptual speed), and DA (indexed by D2DR BP_ND_ in striatum, hippocampus and cortex). This method enables a data-driven characterization of associations between cognitive performance and D2DR availability without relying on arbitary a priori cut-offs, or assuming a consistent linear relationship across the full sample. Four latent profile models with a varying number of classes (1–4) were initially conducted. Model comparison indicated that a model with three classes best described the whole sample (lowest Bayesian information criterion for this model). These three classes were characterized by (1) above-sample mean cognitive performance and D2DR availability (Class 1; n = 99), (2) low cognitive performance and low D2DR availability (Class 2; n = 42), and 3) low cognitive performance coupled with high D2DR availability (Class 3; n = 40). Here, participants were allocated to each of these three subgroups based on their most likely class membership to examine whether the patterns observed for mean-level cognitive performance extend to within-person variability in cognition. Data on IIV did not feature in the previous study.

### Statistical analyses

PET data were missing for one individual, data on WMHs for 8 individuals, DTI data for 3 individuals, and in-scanner n-back data for one individual. Furthermore, extreme outliers (> 3 times the interquartile range below/above the first/third quartile) on behavioral and brain measures were excluded from analyses of the respective measure, leading to exclusion of two individuals from the PET data, three individuals from the WMH data, one individual from the volumetric data, one individual from the finger-tapping task, and two individuals from the DTI data. Participants whose task accuracy was below chance (n = 12 for the n-back task^89^; n = 2 for the perceptual-speed tasks), suggesting misunderstanding of task instructions, were further excluded from analyses of the respective task. The exact sample size for each analysis is displayed in Table 1 and Supplementary Tables S2–S5.

**Table 1:** Mean performance (SD) in the perceptual speed and 1-back tasks.

	Accuracy	Mean RT (s)	ISD RT (s)
Number comparison (n = 179)	0.97 (0.03)	1.70 (0.34)	0.41 (0.13)
Verbal comparison (n = 179)	0.96 (0.04)	1.91 (0.44)	0.56 (0.17)
Figure comparison (n = 179)	0.87 (0.06)	3.61 (0.73)	1.24 (0.35)
1-back (n = 168)	0.84 (0.11)	0.87 (0.11)	0.20 (0.03)

RT data for each task were first preprocessed to exclude extremely fast (< 100 ms for finger-tapping; < 300 ms for the perceptual-speed and 1-back tasks) and slow (> 500 ms for finger-tapping; > 5 s for the numerical-speed and verbal-speed tasks) responses, likely reflecting erroneous responses (e.g., mistaken key press, interruption of task performance)^3^. These cut-offs were determined based on the examination of raw RT distributions across participants for each task, and resulted on average 0.10% to 1.30% of trials being excluded across tasks. For each task, IIV in RT was then computed as the ISD of participants’ RTs across trials, including only correct trials for the perceptual speed and 1-back tasks. For finger tapping, total ISD RT and mean RT reflected the average of the left- and right-hand estimates, and for perceptual speed the average of block-specific estimates. For 1-back, ISD RT and mean RT were calculated across all available trials due to low trial numbers per block. To obtain a single measure of IIV in perceptual speed, a z-standardized composite score was computed that reflected the average ISD RT across the numerical, verbal, and figural-speed tasks. Composite scores were similarly formed for mean RT and accuracy (proportion correct) in the perceptual-speed tasks.

Statistical analyses were performed with SPSS Statistics for Windows Version 25. Data on WMH volume were log-transformed, and perceptual speed accuracy data arcsine-transformed, after which all variables followed an approximate normal distribution (skewness − 0.99 to 0.96; kurtosis − 0.53 to 1.83). Partial Pearson’s correlations were computed to investigate the relationship of brain integrity to behavior. In all analyses of brain-behavior relationships, sex and education were adjusted for, with differences in mean RT further adjusted for in the analyses concerning IIV. Participant age was not added as a covariate due to the age-homogenous nature of the sample. Bias-corrected and accelerated bootstrap 95% confidence intervals (CIs) were computed for all correlations on the basis of 5000 samples. Subgroup differences in RT variability and mean RT were assessed with one-way ANOVAs and ANCOVAs, followed by planned contrasts. Alpha-level was set to *p* < 0.05 (two-tailed, uncorrected) for all analyses.

## Results

### IIV in perceptual speed and 1-back

Performance (accuracy, mean RT, and ISD RT) in each task is displayed in Table 1. Across participants, IIV in RT correlated positively with mean RT in both perceptual speed (composite of numerical, verbal, and figural comparison), *r* = 0.86, CI [0.82, 0.89], *p* < 0.001, and 1-back, *r* = 0.29, CI [0.14, 0.43], *p* < 0.001, indicating that more variable individuals were also generally slower to respond across the tasks. For 1-back, ISD RT correlated negatively with performance accuracy, *r* = − 0.47, CI [− 0.58, − 0.35], *p* < 0.001, with more variable individuals displaying poorer memory performance. Interestingly, for the perceptual-speed tasks, positive correlations between performance accuracy and both ISD RT, *r* = 0.22, CI [0.07, 0.37], *p* = 0.003, and mean RT, *r* = 0.23, CI [0.08, 0.37], *p* = 0.002, were observed, suggesting a speed-accuracy trade-off in these tasks. Both the correlation between ISD RT and accuracy (*p* = 0.575) and between mean RT and accuracy (*p* = 0.252) were non-significant when mean RT, or ISD RT, were added as covariates, respectively, suggesting a shared relationship to accuracy.

Aligning with prior findings indicating consistent between-person differences in IIV across cognitive tasks^56^, we observed a positive correlation between the estimates of IIV computed from the perceptual-speed tasks, and the 1-back task, *r* = 0.20, CI [0.05, 0.37], *p* = 0.009, although most of the variance in each measure was still task-specific. Similarly, perceptual speed mean RT correlated with 1-back mean RT, *r* = 0.37, CI [0.24, 0.50], *p* < 0.001. Between-person differences in ISD RT or mean RT in the finger-tapping control task were not significantly associated with ISD RT or mean RT in perceptual speed (*p*s > 0.448) or 1-back (*p*s > 0.124), suggesting that differences in basic motor function were not a major determinant of individual differences in response time in the tasks involving a cognitive component.

### IIV and brain integrity across the whole sample

We next examined whether between-person differences in IIV were related to individual differences in D2DR availability, as well as grey-, and white-matter integrity, across the whole sample (Supplementary Table S2). Partial correlations adjusting for sex, education, and mean RT, indicated no significant relationships between frontal or striatal D2DR BP_ND_ and IIV in perceptual speed (*p*s > 0.461) across the whole sample. Similarly, in 1-back, ISD RT was not significantly associated with D2DR BP_ND_ in the striatum (*p* = 0.245), although a marginal negative correlation was present in the frontal cortex across the whole sample, *r* = − 0.15, CI [− 0.28, − 0.01], *p* = 0.060. Furthermore, we did not observe significant associations between IIV and frontal or striatal grey-matter volume (*p*s > 0.186), the number or volume of WMHs (*p*s > 0.072), or DTI-derived measures of mean FA (*p*s > 0.197) and MD (*p*s > 0.532) across white-matter tracts connecting the two frontal hemispheres, and the frontal cortex to the parietal, temporal and occipital cortices. As for IIV, individual differences in mean RT were not significantly associated with D2DR availability (*p*s > 0.243), grey-, or white-matter integrity (*p*s > 0.284) across the tasks examined (Supplementary Table S3).

### IIV and D2DR availability in the subgroups

**Figure 1:**
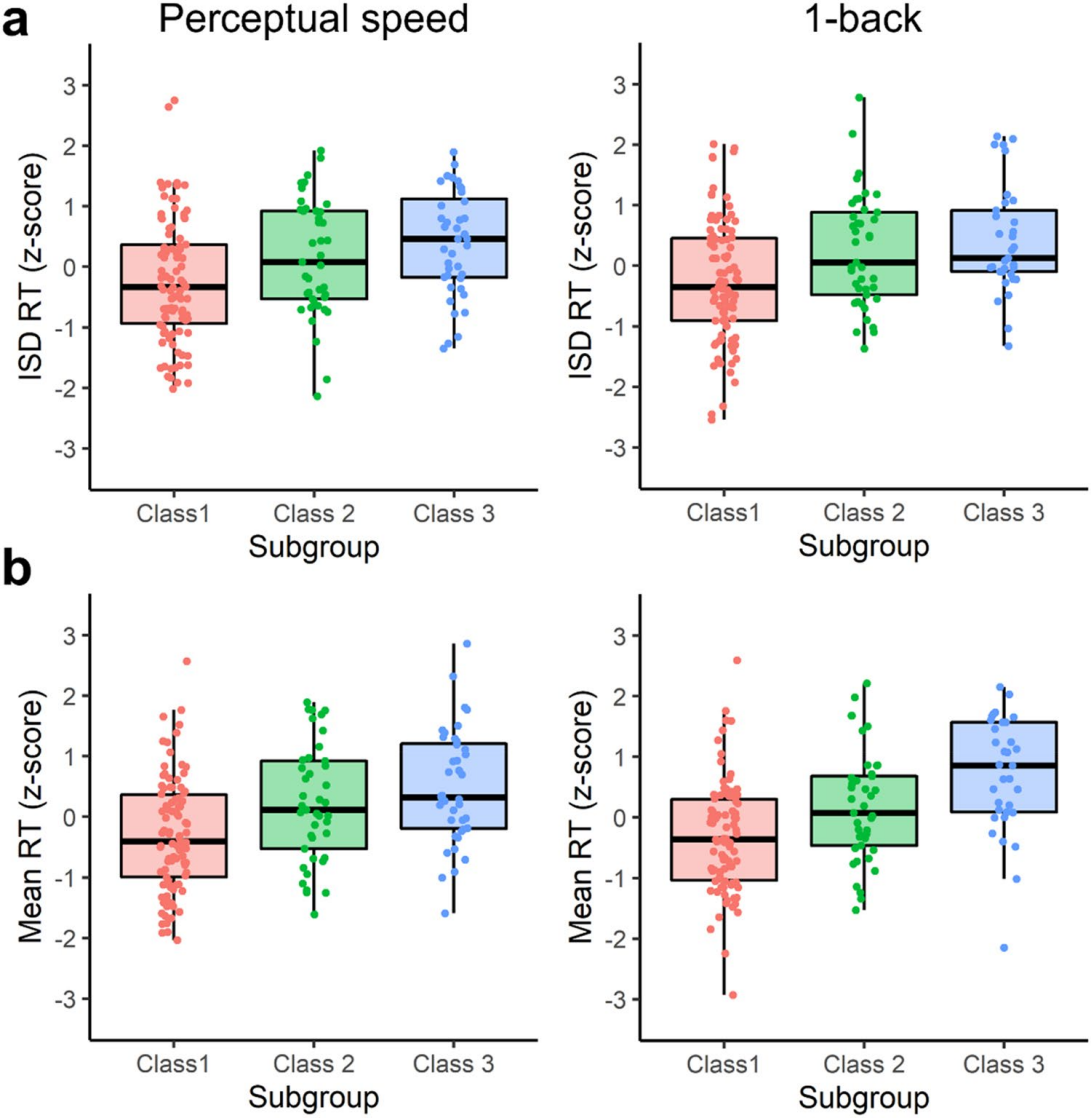
Intra-individual variability in reaction time (RT) (**a**), and mean RT (**b**) in each sample subgroup (Class 1 = high cognition, high D2DR availability; Class 2 = low cognition, low D2DR availability; Class 3 = low cognition, high D2DR availability^60^). Boxplots display the median and upper and lower quartile, and error bars the largest/smallest value within the 1.5 interquartile range from the upper/lower quartile.

Given prior evidence for within-sample heterogeneity in the cognition-D2DR relationship^60^, we next investigated associations between D2DR availability and IIV in the three previously-identified sample subgroups characterized by (1) high cognitive performance, high D2DR availability, (2) low cognitive performance, low D2DR availability, and (3) low cognitive performance, but high D2DR availability. We first examined whether, in addition to mean-level cognitive performance^60^, within-person variability in cognition differed between these subgroups (Fig. 1). A significant main effect of subgroup on IIV was observed for both perceptual speed, *F*(2, 176) = 6.69, *p* = 0.002, *η*_*p*_^2^ = 0.07, and 1-back, *F*(2, 165) = 7.69, *p* = 0.001, *η*_*p*_^2^ = 0.09. In both tasks, Class 2 (*p* = 0.034 for perceptual speed; *p* = 0.006 for 1-back) and Class 3 (*p* = 0.001 for perceptual speed; *p* = 0.001 for 1-back) exhibited higher IIV in comparison to Class 1. For 1-back, *F*(2, 164) = 3.59, *p* = 0.030, *η*_*p*_^2^ = 0.04, but not for perceptual speed (*p* = 0.831), these differences persisted after controlling for mean RT. Indeed, mean RT similarly differed across the subgroups for both perceptual speed, *F*(2, 176) = 11.11, *p* < 0.001, *η*_*p*_^2^ = 0.11, and 1-back, *F*(2, 165) = 16.30, *p* < 0.001, *η*_*p*_^2^ = 0.17. Class 2 (*p* = 0.004) and Class 3 (*p* < 0.001) displayed higher mean RT than Class 1 in both tasks. For 1-back, higher mean RT was also observed in Class 3 in comparison to Class 2 (*p* = 0.014).

**Figure 2:**
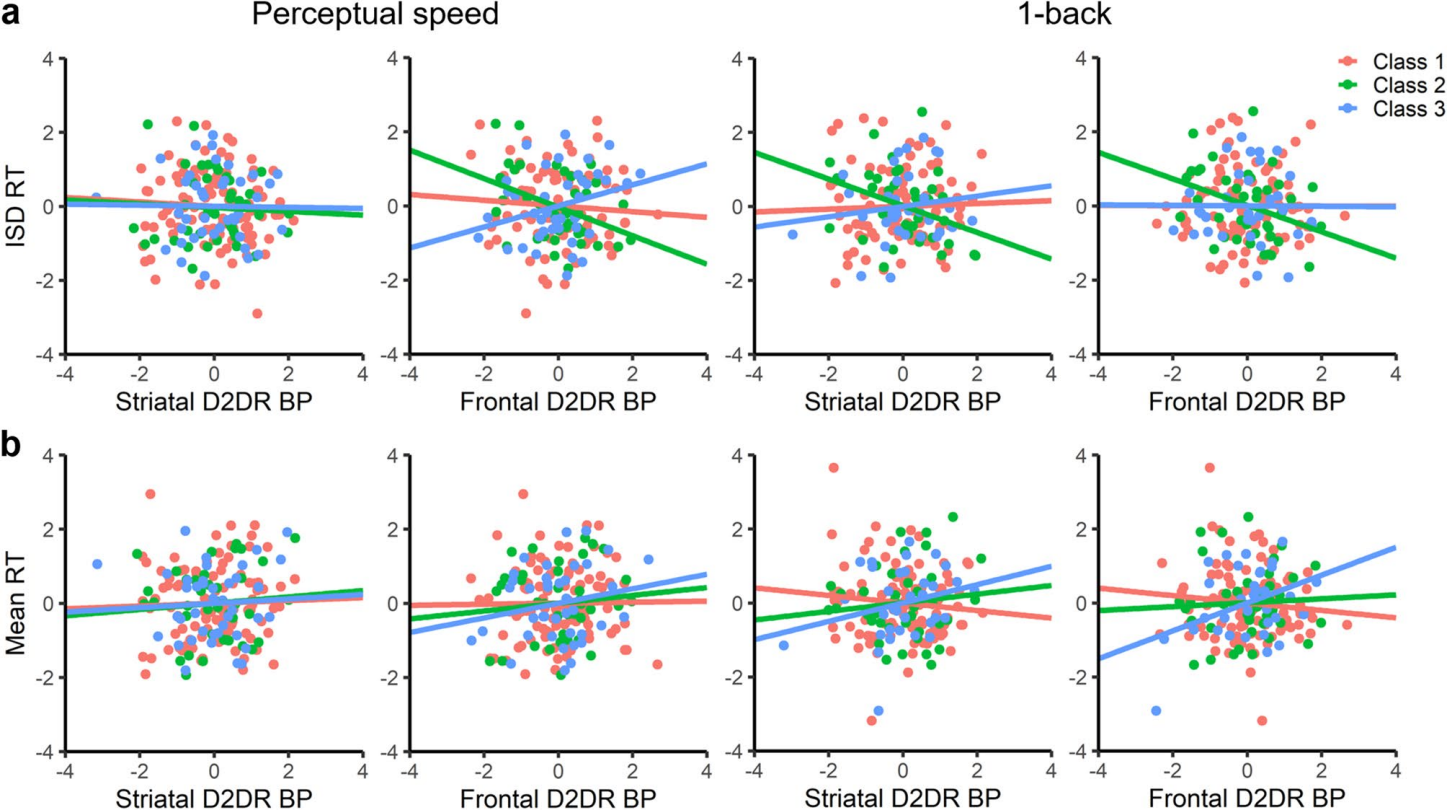
Relationships between fronto-striatal D2DR availability and intra-individual variability in reaction time (RT) (**a**), and mean RT (**b**), colored by subgroup membership (Class 1 = high cognition, high D2DR availability, Class 2 = low cognition, low D2DR availability, Class 3 = low cognition, high D2DR availability^60^). Figures display standardized residuals of each variable after controlling for sex, education, and mean RT (**a**), or sex and education (**b**) within each subgroup.

We next explored whether the increased levels of IIV observed in Classes 2 and 3 may be related to variation in D2DR availability within these subgroups (Fig. 2 and Supplementary Table S4). For Class 2, the subgroup characterized by low cognitive performance and low D2DR availability, we observed frontal D2DR BP_ND_ to correlate negatively with IIV in perceptual speed, *r* = − 0.39, CI [− 0.65, − 0.06], *p* = 0.019, and 1-back, *r* = − 0.35, CI [− 0.61, − 0.05], *p* = 0.034. A negative correlation between striatal D2DR BP_ND_ and IIV was further detected for the 1-back task in this subgroup, *r* = − 0.36, CI [− 0.66, − 0.04], *p* = 0.032, but not for perceptual speed (*p* = 0.765). Similar correlations between D2DR availability and IIV were still observed in Class 2 for both perceptual speed (frontal: *r* = − 0.42, CI [− 0.69, − 0.10], *p* = 0.011) and 1-back (striatal: *r* = − 0.34, CI [− 0.66, 0.01], *p* = 0.046; frontal: *r* = − 0.34, CI [− 0.60, − 0.01], *p* = 0.046) after controlling for IIV in the finger-tapping task, suggesting that these associations were not driven by differences in basic motor variability. Indeed, finger tapping IIV was unrelated to D2DR BP_ND_ in the striatum (*p* = 0.439) and the frontal cortex (*p* = 0.269) in Class 2.

In contrast, no significant associations between D2DR availability and IIV were observed in the other two subgroups (*p*s > 0.088), although we note that the magnitude of correlations between Class 2 and the other two subgroups did not significantly differ in all cases (Supplementary Table S4). For mean RT, a positive correlation between 1-back mean RT and frontal D2DR availability was observed in Class 3, the subgroup characterized by low cognitive performance, but high D2DR availability, *r* = 0.38, CI [− 0.12, 0.68], *p* = 0.037. However, the bootstrap 95% confidence intervals for this correlation overlapped with zero. No further associations between mean RT and D2DR availability were observed in any of the subgroups (*p*s > 0.178; Fig. 2 and Supplementary Table S5). Given evidence for reduced brain integrity beyond the DA system in the low-performing subgroups^60^, we further explored whether individual differences in structural brain integrity were associated with IIV in these groups. There was a positive association between 1-back IIV and WMH volume in Class 3, *r* = 0.40, CI [0.06, 0.66], *p* = 0.040, but no other evidence for associations between grey- or white-matter integrity and IIV in Class 2 (*p*s > 0.151) or Class 3 (*p*s > 0.131).

## Discussion

We examined the role of D2DR availability, as well as grey- and white-matter integrity in within-person variability in cognition using a large sample of healthy older adults participating in the COBRA study^61^. Across the whole sample, no significant associations between brain integrity and IIV were observed. However, within-person variability in cognition was significantly increased in two previously-identified subgroups of individuals displaying low cognitive performance. In one of these subgroups, where such decreases were coupled with reduced subcortical and cortical D2DR availability, we observed frontal D2DR availability to correlate negatively with IIV in both perceptual speed and 1-back, and striatal D2DR availability with 1-back IIV. These results extend previous findings of between-person heterogeneity in the relationship between D2DR and cognitive performance^60^, and suggest that the influence of D2DR availability on within-person variability in cognition may be most easily disclosed in individuals with compromised DA integrity.

The current finding of negative relationships between D2DR availability and cognitive variability in a subgroup of older individuals with reduced D2DR levels aligns with prior accounts suggesting individual differences in brain integrity to have an observable impact on behavior once neural resources fall below a critical threshold^58,59^. For instance, symptoms of Parkinson’s disease are known to emerge following extensive nigrostriatal DA system depletion^90^. In healthy aging, hippocampal volume shrinkage below a critical threshold has been linked to larger episodic-memory decline and increased prefrontal activation levels^91^. Previous studies have also typically observed stronger brain-behavior correlations in older compared to younger adults^22,58^, often taken to reflect age-related decline in brain integrity. Indeed, given the evidence for age-related decreases in both striatal and extrastriatal D2DR levels^92–95^, and for associations between pre- and postsynaptic DA markers^96,97^, it is possible that the low D2DR availability in Class 2 may, at least partly, reflect deterioration of the DA system. However, due to the cross-sectional nature of the current analyses, we cannot distinguish such changes from more stable inter-individual differences in D2DR availability.

In contrast to IIV, we did not observe reliable correlations between mean RT and D2DR availability, aligning with prior findings indicating DA receptor availability to be more consistently linked to RT variability than to mean response speed^55,56^, as well as with behavioral observations indicating IIV to be a more important predictor of cognitive change than mean RT^13^. The current association between D2DR availability and cognitive variability is also consistent with evidence implicating DA in modulating the fidelity and dynamics of neural signalling^47,49,52,53^. In contrast to behavior, moment-to-moment variability in certain measures of brain function, such as fluctuation in fMRI-assessed blood-oxygen-level-dependent (BOLD) signal, has been observed to exhibit age-related decreases^98,99^, and to be negatively associated with behavioral variability^100^. Pharmacological manipulation of DA levels has been shown to boost BOLD variability in older age, with such increases further linked to decreased behavioral variability (in a drug-order-dependent manner)^49^. Future studies should assess whether potential effects of DA on moment-to-moment variability at the neural level mediate the association between DA and behavioral variability observed here and in previous studies^55,56^.

Moreover, we observed similar relationships between D2DR availability and cognitive variability in Class 2 after controlling for differences in finger tapping IIV, suggesting that these associations were not driven by differences in basic motor functions. Thus, our findings align with accounts proposing fluctuations in the efficacy of higher-order, executive control processes to underpin within-person variability in behavior^8,35^. In particular, dopaminergic modulation of fronto-striatal circuits is thought to be important for goal-oriented behavior and cognitive control, balancing the demands between stability and flexibility^33,34^. Prefrontal DA may support the stable maintenance of information in working memory in the face of distraction, whereas striatal DA has been implicated in gating new information into working memory and updating working-memory representations^33,101–103^. We observed IIV in both perceptual speed and 1-back to correlate with frontal D2DR availability in Class 2, whereas a significant association between striatal D2DR availability and IIV was observed in the 1-back task only, which may reflect greater demands on updating posed by this task in comparison to the perceptual-speed tasks. Further, the current findings are consistent with prior PET studies that have linked both pre- and post-synaptic markers of fronto-striatal DA to performance on tasks taxing executive control^104–113^.

Interestingly, prior studies have demonstrated both low and high DA levels to be associated with cognitive impairments, resulting in an inverted-U-shaped association between DA and cognition^33^. Moreover, computational modelling suggests both excessive and insufficient DA to decrease neural signalling fidelity, potentially impacting the stability of behavioral performance^57^. Despite increased levels of cognitive variability, we did not detect significant associations between individual differences in D2DR availability and IIV in Class 3, the subgroup characterized by low cognitive performance and high D2DR availability. This subgroup has lower education levels in comparison to the rest of the sample, as well as reduced structural and functional brain integrity in comparison to Class 1, and in some instances also to Class 2^60^, suggesting that factors beyond DA may contribute to increased IIV in this group. We observed a positive relationship between WMH volume and 1-back IIV in Class 3, but no other evidence for associations between structural brain integrity and IIV in this subgroup. Another possibility is that the high D2DR availability observed in this group could be linked to low endogenous DA levels^60^. Specifically, it is possible that high D2DR levels in this group could reflect receptor upregulation in response to DA degeneration^114,115^, or increased ligand affinity due to reduced competition with endogenous DA^83,116,117^, thereby not accurately capturing integrity of the DA receptor system.

Furthermore, apart from the association between WMH volume and 1-back IIV in Class 3, we did not observe significant relationships between grey- or white-matter integrity and IIV in any of the tasks examined. Many prior studies have demonstrated a link between white-matter integrity and IIV^19,22–24^, whereas results on the role of grey matter in IIV are less consistent^19,21,118^. Prior findings from the COBRA study have linked variation in both grey- and white-matter integrity to differences in mean-level cognitive performance^81,119^. Thus, the current findings question the sensitivity of RT variability, at least as measured in relatively low demanding perceptual-motor or cognitive tasks, as assessed here and in many prior studies^5^, to individual differences in structural brain integrity in healthy aging. It is possible that more demanding cognitive tasks, typically resulting in larger age-related IIV increases^8,55^, would have resulted in a different pattern. While the low-performing subgroups examined here also exhibited reduced structural brain integrity^60^, we note that the subgroup classification was not based on grey-, or white-matter integrity. Therefore, we cannot exclude the possibility that a significant influence of individual differences in grey- or white-matter integrity on IIV could be detectable in certain subgroups of older individuals that display markedly reduced structural integrity of task-relevant brain circuits. Moreover, while our current analyses regarding subgroup differences in IIV provide further support for the previous data-driven characterization of between-person differences in cognition-D2DR relationships within the COBRA sample^60^, this approach should be considered exploratory and awaits further confirmatory studies. Indeed, we note that the correlations between D2DR availability and behavioral variability observed in Class 2 would not survive a strict Bonferroni correction for multiple comparisons.

In conclusion, the current results suggest a role for fronto-striatal DA in cognitive variability in individuals with reduced DA integrity, highlighting the benefits of large-scale studies for delineating between-person heterogeneity in brain-behavior associations in aging. Longitudinal evidence is required to assess the degree to which such relationships may emerge with age-associated deterioration of the DA system, or reflect more stable individual differences in DA-system integrity.

## Supplementary Information

Supplementary Information.

## Data Availability

The datasets generated and analyzed during the current study are available from the corresponding author on reasonable request.
