# Supplementary material for: Fronto-striatal dopamine D2 receptor availability is associated with cognitive variability in older individuals with low dopamine integrity
Source: Sci Rep. 2021 Oct 26;11:21089. doi: 10.1038/s41598-021-00106-y (PMC8548594; doi:10.1038/s41598-021-00106-y)
Supplement: Supplementary file 1 — Supplementary Information. [file 41598_2021_106_MOESM1_ESM.pdf]

Supplementary information

Fronto-striatal dopamine D2 receptor availability is associated with cognitive variability in older individuals with low dopamine integrity

Saana M. Korkki[1]*, Goran Papenberg[1], Nina Karalija[2,3], Douglas D. Garrett[4,5], Katrine Riklund[2,3], Martin Lövdén[6], Ulman Lindenberger[4,5], Lars Nyberg[2,3,7], Lars Bäckman[1]

[1]Aging Research Center, Karolinska Institute and Stockholm University, Stockholm, Sweden

[2]Department of Radiation Sciences, Diagnostic Radiology, Umeå University, Umeå, Sweden

[3]Umeå Center for Functional Brain Imaging, Umeå University, Umeå, Sweden

[4]Center for Lifespan Psychology, Max Planck Institute for Human Development, Berlin, Germany

[5]Max Planck UCL Centre for Computational Psychiatry and Ageing Research, Berlin, Germany and London, UK

[6]Department of Psychology, University of Gothenburg, Gothenburg, Sweden

[7]Department of Integrative Medical Biology, Umeå University, Umeå, Sweden

*Corresponding author

Supplementary information

As illustrated in Figure S1, the response time limitations imposed in the n-back task (maximum response time of 1.5 s) resulted in a truncated distribution of participants' response times for the higher-load conditions. In contrast to load 1, estimates of ISD RT and mean RT did not significantly correlate across participants for load 2, $r = .11$, $p = .150$, or load 3, $r = .00$, $p = .997$. Indeed, when examining only the slowest quartile of participants in each condition, we observed a significant negative association between ISD RT and mean RT for load 2, $r = -.31$, $p = .047$, and load 3, $r = -.48$, $p = .001$. Given the typically positive correlations between mean RT and ISD RT[1], this suggests that within-person variability in response time may not have been accurately captured at the higher load conditions, especially for individuals with slower response speed. Thus, our main analyses focused on the 1-back condition, but we note that no significant correlations between IIV and D2DR availability (ps > .297), or grey- and white-matter integrity (ps > .102) were detected for the 2-back or 3-back conditions across the whole sample.

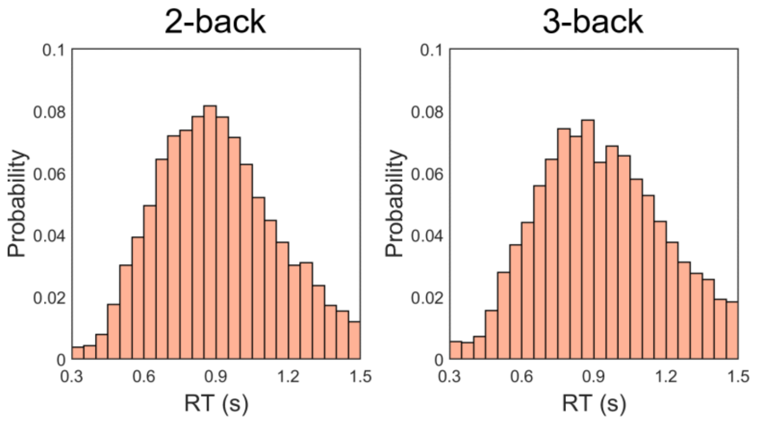

Figure S1. Distribution of participants' response times across correct trials in the 2-back and 3-back conditions of the in-scanner working memory task.

Table S1. Mean ^{11}C-raclopride BP_{ND} (SD) across striatal and frontal subregions (n = 178).

Region	Mean (SD)
Caudate	2.18 (.31)
Putamen	3.35 (.27)
Superior frontal gyrus	.16 (.05)
Middle frontal gyrus	.20 (.05)
Inferior frontal gyrus	.21 (.04)

Table S2. Partial correlations between ISD RT and D2DR availability (n = 176 for perceptual speed; n = 165 for 1-back), grey-matter volume (n = 178 for perceptual speed; n = 167 for 1-back), white matter hyperintensity burden (n = 168 for perceptual speed; n = 158 for 1-back), and white matter microstructure (n = 174 for perceptual speed; n = 165 for 1-back) across the whole sample, controlling for sex, education, and mean RT. Bootstrap 95% confidence intervals are displayed in brackets, p-values uncorrected.

	Perceptual speed			1-back		
	r	CI	p	r	CI	p
D2DR availability						
Striatum	-.03	[-.18, .12]	.670	-.09	[-.25, .07]	.245
Frontal cortex	-.06	[-.21, .11]	.462	-.15	[-.28, -.01]	.060
Grey matter volume						
Striatum	.07	[-.08, .20]	.395	-.08	[-.22, .06]	.285
Frontal cortex	.10	[-.07, .25]	.198	-.10	[-.24, .04]	.187
White matter lesions						
Lesion number	-.02	[-.17, .13]	.822	-.01	[-.15, .13]	.915
Lesion volume	.03	[-.10, .17]	.668	.15	[.00, .28]	.073
DTI						
FA SLF SFOF CC	.10	[-.07, .25]	.217	.10	[-.06, .26]	.198
MD SLF SFOF CC	-.05	[-.20, .11]	.533	.02	[-.13, .17]	.813

Table S3. Partial correlations between mean RT and D2DR availability (n = 176 for perceptual speed; n = 165 for 1-back), grey matter volume (n = 178 for perceptual speed; n = 167 for 1-back), white matter hyperintensity burden (n = 168 for perceptual speed; n = 158 for 1-back), and white matter microstructure (n = 174 for perceptual speed; n = 165 for 1-back) across the whole sample, controlling for sex and education. Bootstrap 95% confidence intervals are displayed in brackets, p-values uncorrected.

	Perceptual speed			1-back		
	r	CI	p	r	CI	p
D2DR availability						
Striatum	.06	[-.10, .21]	.431	.02	[-.11, .15]	.770
Frontal cortex	.09	[-.07, .24]	.244	.04	[-.12, .19]	.609
Grey matter volume						
Striatum	-.03	[-.19, .13]	.670	.08	[-.05, .22]	.317
Frontal cortex	-.03	[-.19, .12]	.704	.00	[-.16, .16]	.977
White matter lesions						
Lesion number	.08	[-.07, .23]	.285	-.01	[-.19, .16]	.879
Lesion volume	.00	[-.16, .17]	.972	-.05	[-.21, .11]	.545
DTI						
FA SLF SFOF CC	.04	[-.12, .20]	.589	-.02	[-.17, .13]	.789
MD SLF SFOF CC	.01	[-.18, .19]	.938	.00	[-.17, .17]	.991

Table S4. Partial correlations between fronto-striatal D2DR BP_{ND} and ISD RT in each subgroup after controlling for sex, education, and mean RT. Bootstrap 95% confidence intervals are displayed in brackets, and significant correlations (p < .05, two-tailed, uncorrected) are highlighted in bold.

	Striatum			Frontal cortex		
	r	CI	p	r	CI	p
Perceptual speed						
Class 1 (n = 97)	-.06	[-.26, .14]	.567	-.08	[-.28, .13]	.463
Class 2 (n = 39)	-.05	[-.40, .30]	.765	**-.39**[b]	**[-.65, -.06]**	**.019**
Class 3 (n = 40)	-.02	[-.30, .28]	.931	.28	[-.01, .54]	.089
WM 1-back						
Class 1 (n = 93)	.04	[-.22, .27]	.723	.00	[-.19, .17]	.978
Class 2 (n = 39)	**-.36**[a,b]	**[-.66, -.04]**	**.032**	**-.35**	**[-.61, -.05]**	**.034**
Class 3 (n = 33)	.14	[-.23, .42]	.462	-.01	[-.34, .31]	.974

Significant difference in correlation magnitudes between [a]Class 2 and Class 1, and [b]Class 2 and Class 3 (*p* < .050).

Table S5. Partial correlations between fronto-striatal D2DR BP_{ND} and mean RT in each subgroup after controlling for sex and education. Bootstrap 95% confidence intervals are displayed in brackets, and significant correlations ($p < .05$, two-tailed, uncorrected) are highlighted in bold.

	Striatum			Frontal cortex		
	r	CI	p	r	CI	p
Perceptual speed						
Class 1 (n = 97)	.04	[-.19, .26]	.717	.01	[-.20, .24]	.893
Class 2 (n = 39)	.08	[-.27, .41]	.622	.10	[-.22, .41]	.540
Class 3 (n = 40)	.06	[-.30, .44]	.726	.20	[-.13, .45]	.238
WM 1-back						
Class 1 (n = 93)	-.10	[-.31, .13]	.335	-.10	[-.26, .08]	.345
Class 2 (n = 39)	.12	[-.18, .38]	.498	.05	[-.25, .40]	.758
Class 3 (n = 33)	.25	[-.12, .51]	.179	**.38**	**[-.12, .68]**	**.037**

References

1. Jensen, A. R. The importance of intraindividual variation in reaction time. *Personality and Individual Differences* **13**, 869–881 (1992).
